# Expression and regulation of *42Sp50* in spotted scat (*Scatophagus argus*)

**DOI:** 10.3389/fgene.2022.964150

**Published:** 2022-08-11

**Authors:** Fei Zhi, Dong-Neng Jiang, Umar Farouk Mustapha, Shao-Xiang Li, Hong-Juan Shi, Guang-Li Li, Chun-Hua Zhu

**Affiliations:** Guangdong Provincial Key Laboratory of Aquatic Animal Disease Control and Healthy Culture, Guangdong Province Famous Fish Reproduction and Breeding Engineering Technology Research Center, Fisheries College of Guangdong Ocean University, Zhanjiang, China

**Keywords:** *Scatophagus argus*, *42Sp50*, 17β-estradiol (E2), promoter analysis, DNA methylation

## Abstract

*42Sp50* is an isoform of the eukaryotic translation elongation factor 1 A (eEF1A) and is vital for fish ovarian development. Spotted scat (*Scatophagus argus*) is a popular marine cultured fish species in Southern Asia and China, and its artificial reproduction is complicated, with a relatively low success ratio in practice. In this study, the *42Sp50* gene was cloned from spotted scat. Tissue distribution analysis showed that *42Sp50* was mainly expressed in the ovary. qRT-PCR showed that *42Sp50* expression levels gradually decreased insignificantly in the ovaries from phase II to IV. Western blot analysis showed that *42Sp50* was highly expressed in the ovary, while it was almost undetectable in the testis. Immunohistochemistry analysis stained *42Sp50* mainly in the cytoplasm of the previtellogenic oocytes in ovaries of normal XX-female and sex-reversed XY-female. Aside from fish and amphibians, *42Sp50* was also identified in some reptile species using genomic database searching. Analyses of the transcriptome data from four different fish species (Hainan medaka (*Oryzias curvinotus*), silver sillago (*Sillago sihama*), Nile tilapia (*Oreochromis niloticus*), and Hong Kong catfish (*Clarias fuscus*)) revealed ovaries biased expression of *42Sp50* in all, similar to spotted scat. While the neighbor genes of *42Sp50* did not show ovary biased expression in the fish species analyzed. Bisulfite Sequencing PCR (BSP) results showed that the DNA methylation level of *42Sp50* promoter was low in ovaries, testes, and muscles. The luciferase reporter assay demonstrated that *Dmrt4* activated *42Sp50* expression in the presence of *Sf1* or *Foxh1*. These results suggest that *42Sp50* may be involved in regulating the early phase oocytes development of spotted scat.

## Introduction

Translation elongation factor (EF) plays an essential role in protein synthesis by transporting the aminoacylated-tRNAs to the ribosome. There are three types of EF in prokaryotes, namely elongation factor thermo unstable (EF-TU), elongation factor thermo stable (EF-TS), and elongation factor G (EF-G). However, two types of EF exist in eukaryotes, including eukaryotic translation elongation factor 1 (eEF1) and eukaryotic translation elongation factor 2 (eEF2). eEF1 is composed of two types of proteins in vertebrates, which include eEF1A and eEF1B. eEF1A, and eEF1B are respectively homologues of prokaryotes EF-TU and EF-TS, while eEF2 is homologue of prokaryotes EF-G. eEF1A forms a compound with GTP and aminoacylated tRNA (aa-tRNA) to transport aa-tRNA to ribosomal site A (Browne and Proud, 2002). eEF1B binds to eEF1A-GDP, accelerating the exchange of GDP bound to eEF1A with exogenous GTP and prompting the formation of eEF1A-GTP (Janssen and Möller, 1988). When peptide bonds are formed, eEF2 promotes the movement of peptide chains from site A to site P of ribosome (Djumagulov et al., 2021).

The number of *eEF1A* subtypes varies among vertebrates species. Two *eEF1A* genes, *eEF1A1* and *eEF1A2*, were reported in human (*Homo sapiens*) (Brands et al., 1986; Knudsen et al., 1993), mouse (*Mus musculus*) (Lee et al., 1994) and chicken (*Gallus gallus*) (Yoshikawa et al., 1984; Wang et al., 1994). Three *eEF1A* genes (*eEF1S*, *eEF1O* and *42Sp50*) were identified in frog (*Xenopus laevis*) (Viel et al., 1991). The number of *eEF1A* gene subtypes is relatively high in fish. In Senegalese sole (*Solea senegalensis*) and Nile tilapia (*Oreochromis niloticus*), five *eEF1A* genes (*eEF1A1a*, *eEF1A1b*, *eEF1A3*, *eEF1A4,* and *42Sp50*) were reported (Infante et al., 2008; Wei, 2011). Four *eEF1A* genes (*eEF1A1a*, *eEF1A1b*, *eEF1A2* and *eEF1A4*) were described in zebrafish (*Danio rerio*) (Idigo et al., 2020)*.* More *eEF1A* genes in fish might be due to the teleosts specific genome duplication (3R) (Hoegg et al., 2004).

The *eEF1A* genes show differential tissue-specific expression patterns in different species. In mammals, *eEF1A1* and *eEF1A2* were differentially expressed in tissues at different development stages, and *eEF1A1* was expressed in more tissue types than *eEF1A2* (Knudsen et al., 1993; Chambers et al., 1998; Kahns et al., 1998). But in the non-mammalian vertebrates, the expression patterns of *eEF1A* genes have not been widely studied. In zebrafish, *eEF1A1a*, *eEF1A1b*, and *eEF1A4* were expressed in the brain, muscle, spleen, intestine, liver, testis, and ovary, while *eEF1A2* was not expressed in the liver (Idigo et al., 2020). In Nile tilapia, *eEF1A1b* was highly expressed in the brain, heart, liver, intestine, kidney and testis, but weakly expressed in the ovary and undetectable in the gill, spleen, head kidney, and muscle. Whereas *eEF1A4* was widely expressed in all tissues, *42Sp50* was specifically expressed in the ovary (Wei, 2011; Chen, 2018). The diverse expression pattern of the *eEF1A* genes in vertebrates suggests differential functions.

*42Sp50* is a special member of the *eEF1A* gene family, first reported in *X. laevis* (Viel et al., 1991). *42Sp50* protein is one component of the ribonucleoprotein particle isolated from the ovary of frog sedimenting at 42 S (Wegnez and Denis, 1979). *42Sp50* has a molecular weight of around 50 kDa, hence the name *42Sp50*. In *X. laevis* and Nile tilapia, *42Sp50* was specifically expressed in the oocytes (Deschamps et al., 1991; Wei, 2011; Chen 2018). The ovarian *42Sp50* gradually increases during the development process, reaching a peak at 60 days after hatching, and then decreases thereafter in Nile tilapia (Wei, 2011). In Japanese medaka (*Oryzias latipes*), *42Sp50* is one of the earliest markers of sex differentiation specifically expressed in oocytes (Kinoshita et al., 2009). While the reason for the ovary biased expression of *42Sp50* remains unclear. Knockout of *42Sp50* blocks the development of oocytes at the primary growth stage leading to abnormal proliferation and differentiation of follicular cells in female Nile tilapia, indicating that *42Sp50* is indispensable for ovarian development in Nile tilapia (Chen, 2018). Further comparative studies are required to understand the transcriptional regulation and functional importance of *42Sp50* in fish.

Spotted scat (*Scatophagus argus*) is a euryhaline subtropical marine fish widely distributed in Indian-Pacific waters (Barry et al., 1993). Spotted scat’s market value is relatively high due to its delicious taste. Although there are reports of artificial reproduction in spotted scat, the efficiency is still low in practice (Mandal et al., 2021). The cultured spotted scat fry were mainly wildly caught in China. This threatened and destroyed the natural resource of spotted scat. Studies focused on the reproduction-related gene in spotted scat might help improve artificial reproduction efficiency. In the previous study, *42Sp50* was found to play an important role in the development of ovaries in Nile tilapia (Chen, 2018). In this study, *42Sp50* was isolated in spotted scat, and its expression was analyzed by quantitative real-time PCR (qRT-PCR), Western blotting, and immunohistochemistry (IHC) during ovary development. In addition, the transcriptional regulation of *42Sp50* was examined. *42Sp50* in spotted scat was specifically expressed in the primary oocytes and regulated by *Dmrt4*, *Sf1,* and *Foxh1 in vitro*. The exclusive expression pattern of *42Sp50* in the primary oocytes enabled it as a good marker gene for further reproduction-related studies in spotted scat. The results provide a basis for the functional study of *42Sp50* in ovarian development in fish.

## Materials and methods

### Experimental fish and sampling

Spotted scats (body weight: 109.0–234.4 g, length: 15.4–16.9 cm) purchased from the Dongfeng market (Zhanjiang, Guangdong, China) were used for cDNA cloning and expression analysis. All fish were anesthetized using 100 mg/L of tricaine methane sulfonate (MS 222, Sigma, St. Louis, MO, United States) and dissected. Tissues used for further studies were excised and snap-frozen in liquid nitrogen and stored at −80°C prior to use. Parts of the excised gonads were fixed in Bouin’s solution for gonad developmental stage characterization and IHC analysis. The tail fins were also collected, stored in 100% ethanol at −20°C for DNA extraction. All experimental fish protocols followed the guidelines and approval of the Administration of Affairs Concerning Experimental Animals for the Science and Technology Bureau of China, and Animal Research and Ethics Committees of Guangdong Ocean University.

The induction of sex reversed XY fish was described in our previous study (Mustapha et al., 2021). Briefly, a batch of about 2-month-old spotted scat (average total length 2 cm) were obtained from the wild in August 2019 from Beihai-China and cultured at the Zhanjiang Donghai Island Cultivation Base (Zhanjiang, Guangdong, China). After 1 week, the fish were equally divided into two groups: the control group and the Estrogen group (E2 group). The E2 group was fed diets containing Estrogen (300 μg/g) while the control group was fed with common diets. Fish were fed three times daily for 3 months, and treatment terminated. The fish were subsequently fed with common diet until sampling. Before sampling, spotted scat were about 2.5 years old. Spotted scat were anesthetized, and the gonads were quickly dissected. Parts were fixed in Bouin’s solution for IHC, and the other part stored in 100% ethanol for DNA extraction. The tail fins were also collected, stored in 100% ethanol at −20°C for DNA extraction.

### Identification of genetic sex of spotted scat

The DNA of spotted scats was extracted by a nucleic acid purification kit (N1173, DONGSHENG BIOTECON, China). After the quality and concentration were detected by 1% agarose gel and Nanodrop 2000 ultramicro nucleic acid protein analyzer (Thermo Scientific), DNA was stored at −20°C until use. The genetic sex of spotted scats was determined by using the sex-linked DNA Marker (*Dmrt1*-Marker-4-F/R) (Huang et al., 2022). Primer sequences are listed in Supplementary Table S1 and the PCR conditions were the same as Huang et al. (2022). *Dmrt1*-Marker-4-F/R amplified only one DNA fragment of 593 bp in female, while two DNA fragments of 593 bp and 693 bp were amplified in male.

### cDNA cloning, gene identification and bioinformatics analysis

Total RNA was extracted from testes and ovaries of adult fish using TRIzol reagent (Invitrogen). The quality and concentration of RNA were detected by 1% agarose gel and Nanodrop 2000 ultramicro nucleic acid protein analyzer (Thermo Scientific). First-strand cDNA synthesis was performed using the PrimeScript^TM^ RT reagent Kit with gDNA Eraser (Takara, China) following the manufacturer’s protocol.

Local BLAST was used to search for the *42Sp50* transcripts from transcriptome sequence data of spotted scat gonads (He et al., 2019) using the *42Sp50* mRNA sequence from Nile tilapia (NCBI accession number: XM_019363060.2) as a query. Primers were subsequently designed from the *42Sp50* sequence obtained from the transcriptome data to amplify the partial cDNA fragment, including the open reading frame (ORF) in spotted scat (Supplementary Table S1). The PCR conditions were as follows: 4 min at 94°C; 30 s at 94°C, 30 s at 53°C, and 2 min at 72°C for 35 cycles; 10 min at 72°C. PCR products were examined on 1.5% agarose gel stained with ethidium bromide. Amplification bands were excised from the gel, purified and cloned into the pEasy-T3 vector (TransGen Biotech, China). Positive clones were screened by colony PCR and sequenced by Sanger sequencing (Sangon, Shanghai, China). The cDNA sequences of *42Sp50* cloned in this study was analyzed using DNASTAR software (http://www.dnastar.com). The ORF and protein sequences were predicted using ORF Finder software (https://www.ncbi.nlm.nih.gov). Sequence homology alignments were performed using MegAlign (part of the DNASTAR software package), and multiple alignments were carried out using ClustalX (http://www.clustal.org/). The whole protein sequences of *42Sp50* from other vertebrate species (the accession numbers are listed in Supplementary Table S2) were downloaded from NCBI (https://www.ncbi.nlm.nih.gov/). The phylogenetic tree was constructed using the neighbor-joining method implemented in MEGA X (https://www.megasoftware.net/). Syntenic analysis was done by using data on Ensembl (https://asia.ensembl.org/index.html), NCBI (https://www.ncbi.nlm.nih.gov/) and Genomicus (https://www.genomicus.bio.ens.psl.eu/genomicus-101.01/cgi-bin/search.pl).

### Tissue distribution of *42Sp50* mRNA

The hypothalamus, pituitary, gill, liver, heart, spleen, kidney, stomach, intestine, gonad, and muscle were dissected from adult male and female spotted scats and were used for tissue distribution analysis. Tissue distribution was performed by reverse transcription-polymerase chain reaction (RT-PCR). The cDNA was diluted 5-fold and subjected to amplification. β*-Actin* was used as an internal control. The PCR condition was as follow: 5 min at 94°C; 30 s at 94°C, 30 s at 60°C, and 30 s at 72°C for 32 cycles (β*-actin* 28 cycles); 10 min at 72°C. The PCR products were separated by electrophoresis on 1.5% agarose gel and visualized with ethidium bromide.

### Expression of *42Sp50* mRNA in ovaries at different stages

Ovaries from adult females at different stages of gonadal development were collected. The gonad developmental phases were determined by histological sectioning and staining, according to Cui et al. (2013). Total RNA was extracted from the gonads and reverse transcription was carried out, as mentioned above. The mRNA levels of *42Sp50* at different stages of gonadal development were detected by qRT-PCR and the conditions were as follows: 1 min at 95°C, 30 s at 95°C, 30 s at 60°C, 30 s at 72°C for 40 cycles (fluorescent data collection). The relative abundance of *42Sp50* mRNA transcripts was evaluated using the formula, R = 2^−ΔΔCt^ (Livak and Schmittgen, 2001). β*-Actin* was used as a reference gene. All determinations were performed in triplicate. Primer sequences used for qPCR are listed in Supplementary Table S1. The amplified fragment length of *42Sp50* and β*-actin* were around 183 and 147 bp, respectively. The specificity of the primers were confirmed by cloning and sequencing.

### Western blot

The whole protein sequence of spotted scat *42Sp50* was used as antigen to prepare the polyclonal antibody in this study. The recombinant *42Sp50* constructs were prepared by cloning the ORF into the expression vector pET16b. With MBP-tag at its N-terminus and His-tag at its C-terminus, the recombinant plasmid was expressed in *Escherichia coli* with isopropyl β-D-l-thiogalactopyranoside (IPTG, 500 μM) induction. Next, the recombinant protein (25–30 μg) was purified using a Ni-NTA super flow cartridge (Qiagen, Germany) and used as an antigen to immunize female rabbits (Japanese white rabbit) three times at 15- day intervals. Ten days after the last immunization, rabbit serum was collected and purified by affinity chromatography on Sepharose 4B Fast Flow Resin (Sigma, Germany). Polyclonal antibodies were confirmed by Western blot analysis. Briefly, total protein was extracted from male (*n* = 3) and female (*n* = 3) gonads (both at phase III) from adult spotted scat and diluted to a final concentration of 20 mg/ml. Western blotting was carried out as described previously (Ru et al., 2020). Antibody against *42Sp50* was diluted at 1:2,500 and the abundance of β-actin (rabbit anti-β-actin, 1:1,000 dilution of AA002, Abiotech co., Ltd. (Jinan, China)) was examined as a loading control. Goat anti-rabbit antibodies conjugated to horseradish peroxidase (1:1,000 dilution of YKCP-202-01, Youke, China) were used as secondary antibodies. Immunoreactive signals were detected with the BeyoECL Plus Kit (Beyotime, China) and then visualized on Tanon 5,200 (China). According to a previous report (Holmseth et al., 2012), an antigen pre-adsorption test was performed as a negative control.

### Immunohistochemistry analysis

As described above, the gonads of the spotted scat at different gonad developmental phases and estradiol (E2) treated fish were collected. Gonads were fixed in Bouin’s solution for 24 h at room temperature, then dehydrated and embedded in paraffin. Tissue blocks were sectioned at 5 μm thickness. IHC was carried out according to the method used by Ru et al. (2020). Antibody against *42Sp50* was diluted to 1:500 and detected by the rabbit anti-goat antibody conjugated with horseradish peroxidase (1:1,000 dilution of YKCP-202-01, Youke, China). The pre-adsorption primary anti-*42Sp50* antibody was also used as a negative control, according to a previous report (Holmseth et al., 2012).

### Transcriptome analysis of the expression of *42Sp50* gene and its adjacent genes in different species

The sequences of *42Sp50* gene and its collinear genes of Nile tilapia and medaka were obtained from NCBI. Through local blast, the expression levels of *42Sp50* gene and its collinear genes were searched in the transcriptome data of spotted scat (He et al., 2019), Nile tilapia (Tao et al., 2013), Hainan medaka (*O. curvinotus*) (Dong et al., 2021), Hong Kong catfish (*Clarias fuscus*) (Lin et al., 2021) and silver sillago (*Sillago sihama*) (Tian et al., 2019). Transcriptome samples of Nile tilapia, medaka and silver sillago were all one female and one male, and the transcriptome samples of spotted scat and Hong Kong catfish were three females and three males, and the average value of the data was taken.

### DNA bisulfite treatment and sequencing

The 3 kb sequence before the *42Sp50* start codon was found in the spotted scat genome as its promoter sequence. The CpG Islands of *42Sp50* promoter were predicted with MethPrimer (https://www.urogene.org/cgi-bin/methprimer/methprimer.cgi). Selected fragment within the 3 kb region was used to design a pair of primers (Supplementary Table S1). The gonads of three wild XY individuals, three wild XX individuals and two XY individuals treated with E2 were selected for DNA extraction, referring to the previous method. Bisulfite modification of spotted scat DNA was performed using the EZ DNA Methylation-Gold™ Kit (D5005, Murphy Ave, Irvine, CA, United States), following the manufacturer’s instructions. The bisulfate-treated DNA was amplified by PCR using the previously mentioned primers, and then the PCR product was ligated into the pEasy-T3 vector (TransGen Biotech, China). For each individual, 7–10 positive clones were sequenced. The Sanger sequencing was carried out by Sangon (Shanghai, China). The sequencing results were compared with spotted scat *42Sp50* gene sequence.

### Transient transfections and luciferase assays

The possible binding sites of *42Sp50* promoter were predicted by PROMO (http://alggen.lsi.upc.es/cgi-bin/promo_v3/promo/promoinit.cgi?dirDB=TF_8.3). The *42Sp50* promoter of spotted scat (3 kb, ligation into *Mlu* I/*Xho* I sites) was generated by PCR and subcloned into the pGL3-basic vector (Promega Corp., Madison, WI). *Sf1*, *Foxh1,* and *Dmrt4* sequence was synthesized by Sangon Biotech and ligated to pcDNA3.1 vector (Invitrogen). The dual-luciferase assay was carried out according to a previous report (Wang et al., 2007). HEK 293 cells cultured at a density of 2  ×  10^4^ cells per well were used for transfection. The reagents used in the experiment include FBS (16140071, Gibco, America), DMEM (SH30022.01, Hyclone, America), streptomycin/penicillin (15070063, Thermofisher, America), Dual-Luciferase Reporter Gene Assay Kit (K801, Biovision, America) and lipo2000 (Invitrogen, America).

### Statistical analysis

All data are expressed as means ± standard deviation (SD). Significant differences in the data among the groups were tested by one-way analysis of variance (ANOVA) with Duncan’s post-hoc test or independent-samples *t*-test using a threshold of *p* < 0.05. All statistical calculations were performed using SPSS 19.0 (SPSS, Chicago, IL, United States).

## Results

### cDNA cloning and sequence analysis of *42Sp50*

**FIGURE 1:**
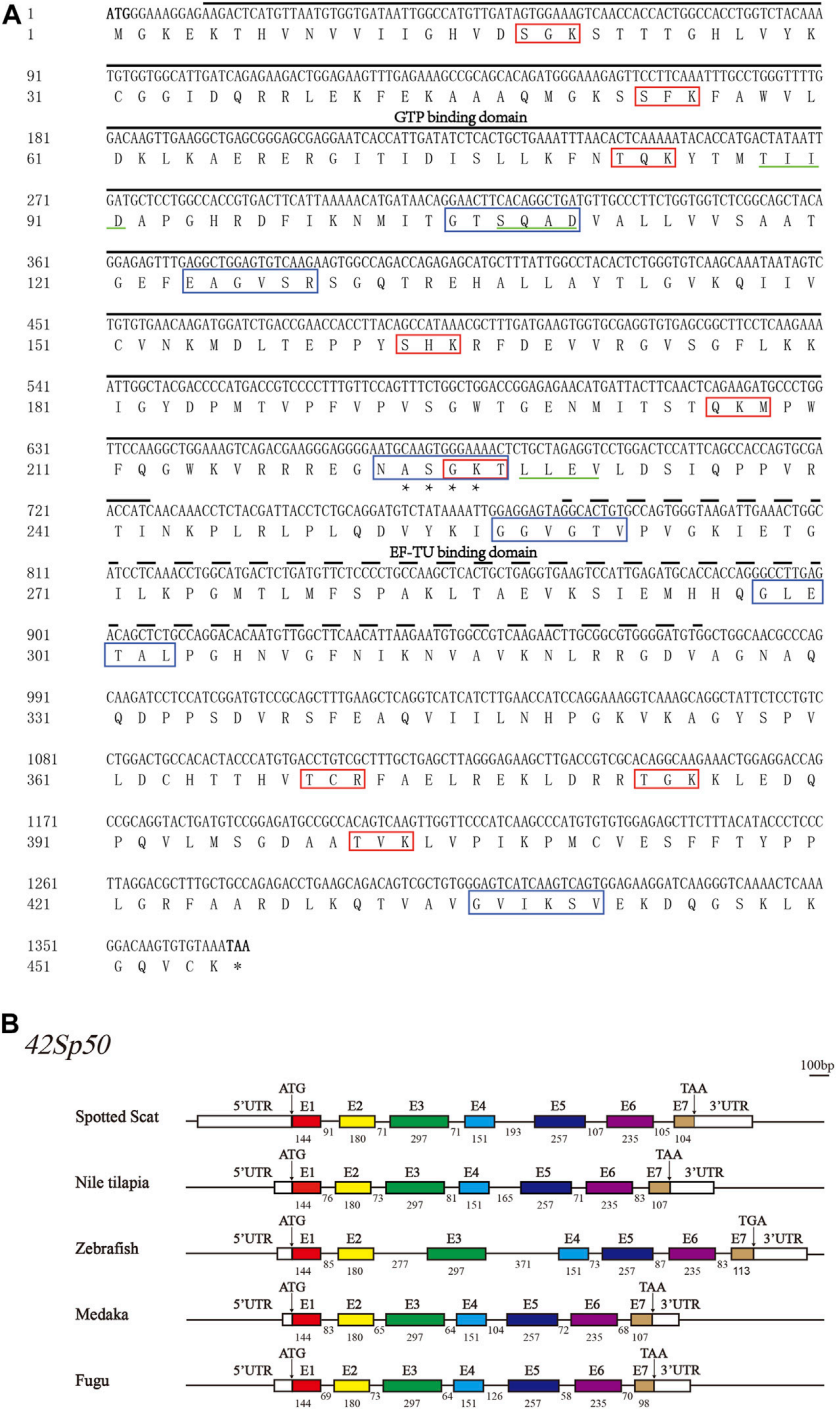
The gene sequence and structure of *42Sp50* in spotted scat (*Scatophagus argus*). **(A)** Nucleic acid sequence and amino acid sequence of spotted scat *42Sp50*. Black solid line, the GTP domain; Dotted line, EF-TU domain; “*”, N-glycosylation site; Red box, Protein kinase C phosphorylation site; Green solid line, Casein kinase II phosphorylation site; Blue box, N-myristoylation sites. **(B)** Schematic diagram of the exon–intron structure of *42Sp50* in bony fish. The white rectangles represent the 5′ and 3′ UTR. Colored boxes represent exons, and horizontal lines between exons represent introns. ATG stands for the start codon and TAA/TGA stands for the stop codon. The number represents the base length. The short solid lines at the top right are scale bars, representing 100 bp.

The partial cDNA of spotted scat *42Sp50* was 1543 bp with 1368 bp ORF which encodes 455 amino acids (NCBI accession number: MT774144) (Figure 1A). The predicted molecular mass was 50.05 kDa, and the isoelectric point was 9.63. Spotted scat *42Sp50* contains seven exons and six introns, similar to other fish (Figure 1B). The sequence analysis showed that *42Sp50* has no transmembrane domain and signal peptide, but with one N-link glycosylation site, nine protein kinase C phosphorylation sites, three casein kinase II phosphorylation sites and six N-myristoylation sites. Additionally, GTP binding domain and EF-TU binding domain were predicted in the sequence (Figure 1A). The structure and sequence of spotted scat *42Sp50* are conserved with other fish species (Supplementary Figure S1).

**FIGURE 2:**
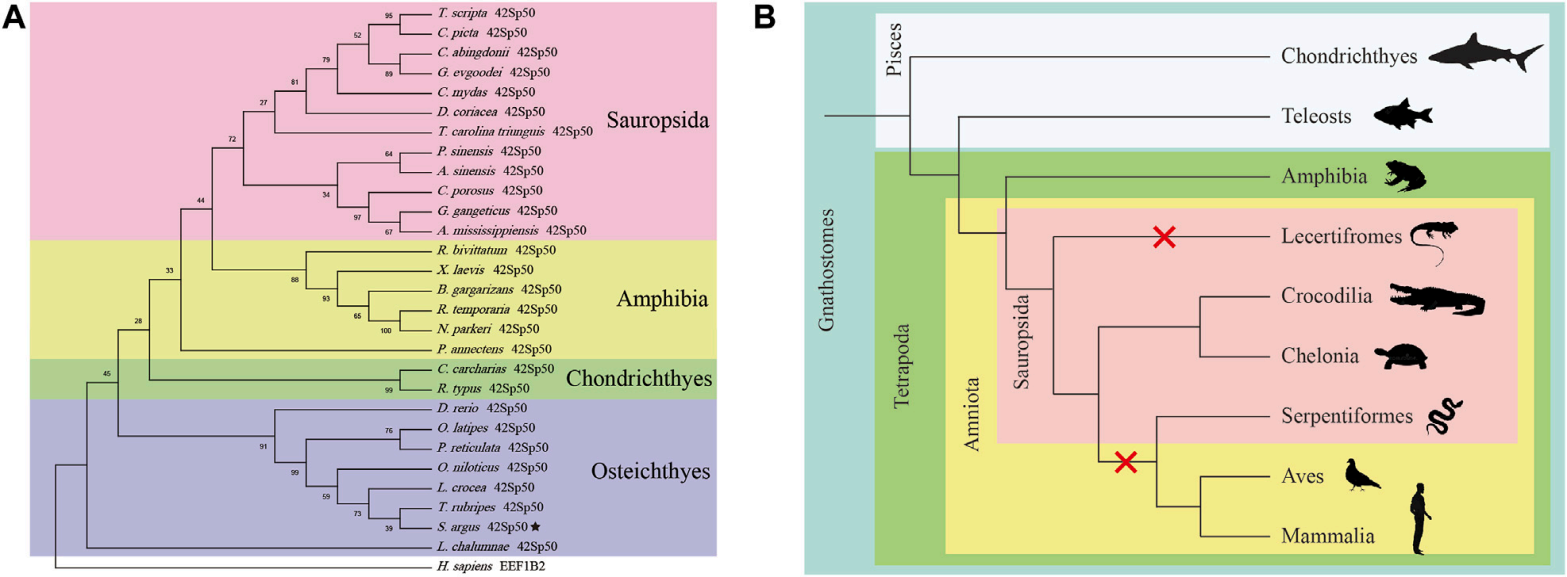
Phylogenetic analysis of *42Sp50* in vertebrates. **(A)** Phylogenetic tree of the 42Sp50 in selected vertebrate species. The tree was constructed using MEGA X and the Neighbor-joining approach. Bootstrap testing was based on 500 replicates. The *42Sp50* genes of spotted scat are denoted by pentagram. Genbank accession numbers of these are provided in Supplementary Table S2. **(B)** The evolution of *42Sp50* in Gnathostomes. The red X signs indicate the events of *42Sp50* losses during the evolution of Lecertifromes, Serpentiformes, Aves and Mammalia.

In this study, *42Sp50* was isolated from the genome database of fish, amphibia and some reptiles. A phylogenetic tree based on the amino acid of *42Sp50* sequences of vertebrates was constructed (Figure 2A) (the accession numbers are listed in Supplementary Table S2). The analysis showed that the sequence found in reptiles was indeed *42Sp50*. It was observed that *42Sp50* of sauropsida, amphibia, chondrichthyes, and osteichthyes were clustered into four distinct branches. *42Sp50* of spotted scat was most closely related to Fugu (*Takifugu rubripes*). However, *42Sp50* was not isolated in all reptile genomes. *42Sp50* was found in chelonia and crocodilia, but not in lecertifromes and serpentiformes. Due to the lack of data, the presence of *42Sp50* is unknown in rhynchocephalia and amphisbaeniformes. In addition, *42Sp50* was not found in aves and mammalia (Figure 2B).

**FIGURE 3:**
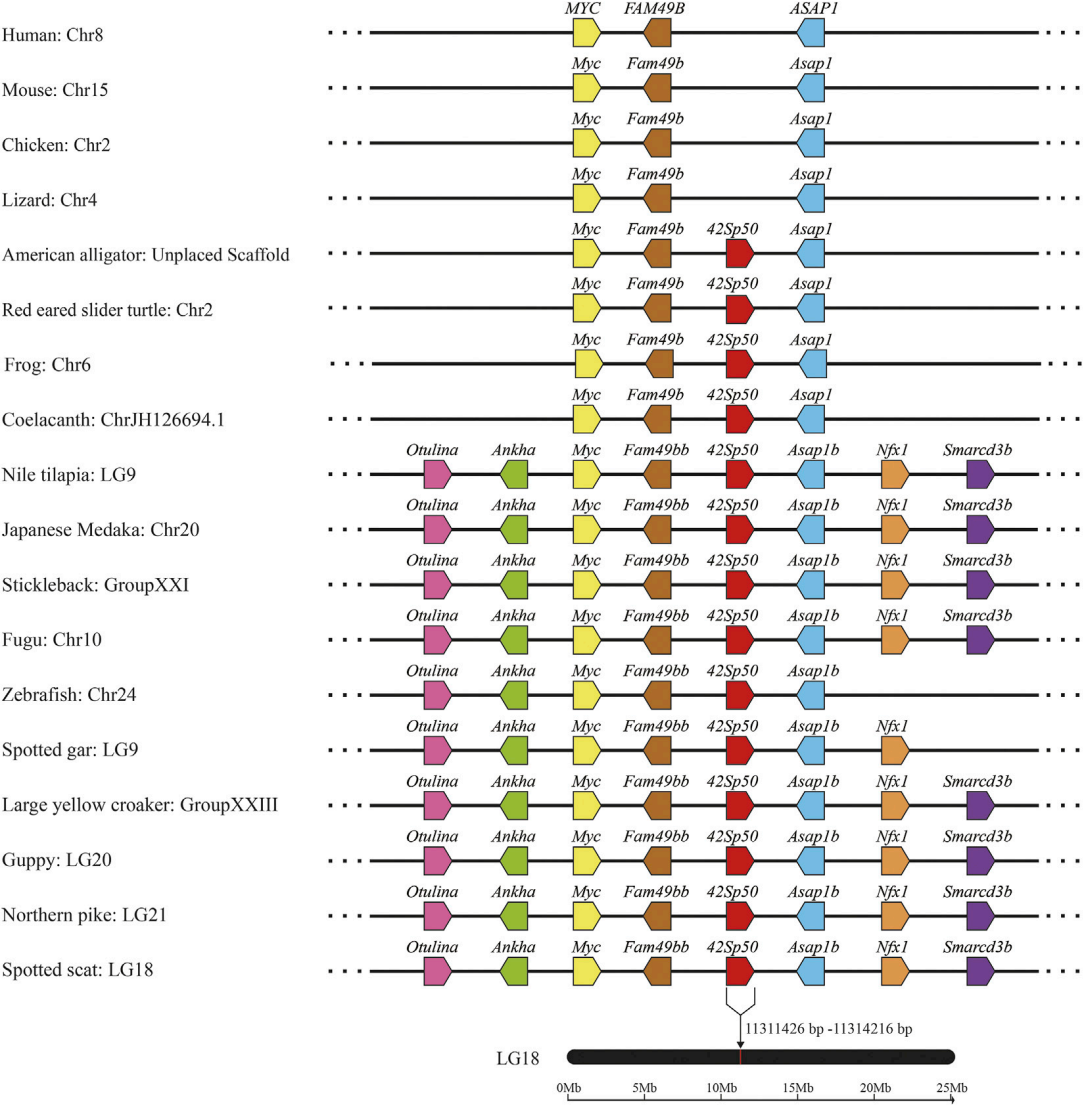
Syntenic analysis of *42Sp5*0 genes from spotted scat using the genomes of 18 selected vertebrate species (human, mouse, chicken, lizard, American alligator, red eared slider turtle, frog, coelacanth, Nile tilapia, Japanese medaka, stickleback, Fugu, zebrafish, spotted gar, large yellow croaker, guppy and northem pike). Genes are represented by pentagons, and the directions of the reading frames are represented by the direction that the pentagon is pointing. Genbank accession numbers of these gene are provided in Supplementary Table S3.

In order to further understand *42Sp50*, synteny analysis was performed. The *42Sp50* gene of spotted scat is located at 11311436-11314216 bp of LG8 chromosome. The results indicated that the adjacent genes of *42Sp50* have a high degree of consistency in fish. Even though *42Sp50* was not found in human, mouse, chicken and lizard (*Anolis carolinensis*), its upstream and downstream genes (e.g., *Myc*, *Fam49b* and *Asap1*) exist in these species (Figure 3). The accession number of genes used in syntenic analysis are listed in Supplementary Table S3.

### Tissue distribution and expression of *42Sp50* mRNA at different gonad developmental phases

**FIGURE 4:**
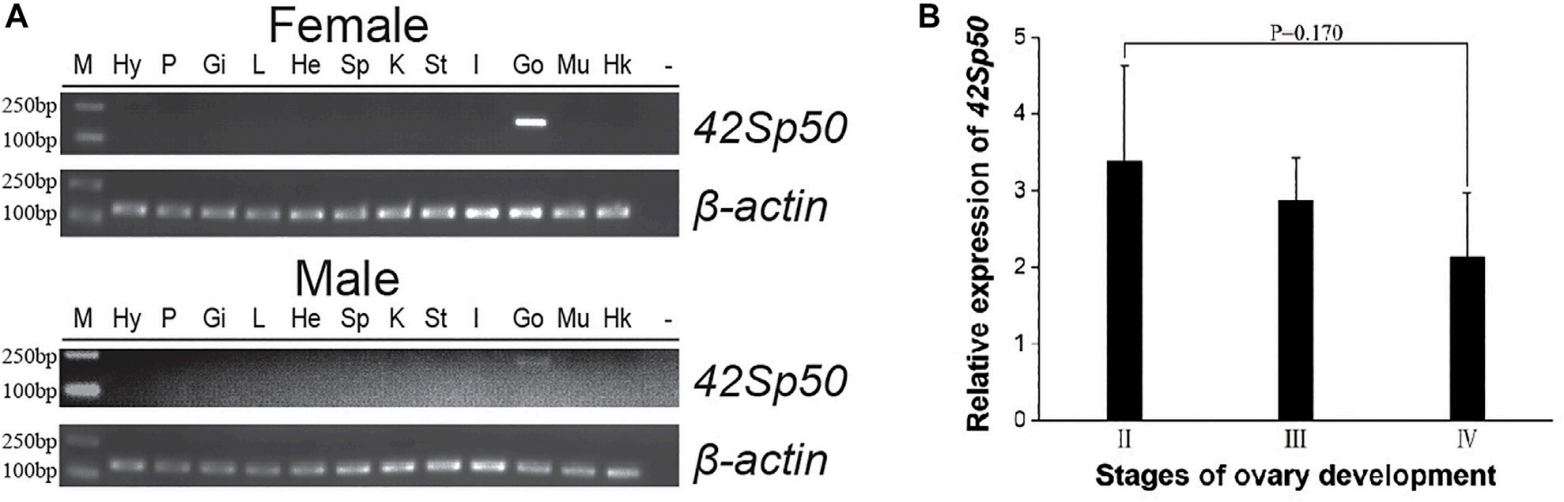
Tissue distribution and expression of *42Sp50* mRNA at different gonad developmental phases. **(A)** Tissue distribution of *42Sp50* in spotted scat. The PCR products for spotted scat *42Sp50* and β*-actin* were 183 bp and 145 bp, respectively. Hy, hypothalamus; P, pituitary; Gi, gill; L, liver; He, heart; Sp, spleen; K, kidney; St, stomach; I, intestines; Go, gonad; Mu, muscle; “−”, negative control; Marker, DS™2000. **(B)** The expression of *42Sp50* in the ovary at different development stages in spotted scat using qRT-PCR. Data are presented as mean ± SEM (*n* = 3).

Tissue expression results showed that *42Sp50* was highly expressed in the female ovaries and was weakly expressed in the testis (Figure 4A). The distinction of ovarian stages is detailed in Cui et al. (2013). The ovaries of stage II, stage III and stage IV of spotted scat were collected, and the *42Sp50* mRNA expression levels were examined by qRT-PCR. The highest expression level of *42Sp50* mRNA was observed in stage II and the lowest in stage IV, but there was no significant difference between the expression levels in each stage (Figure 4B).

### *42Sp50* protein expression in gonads by western blot

**FIGURE 5:**
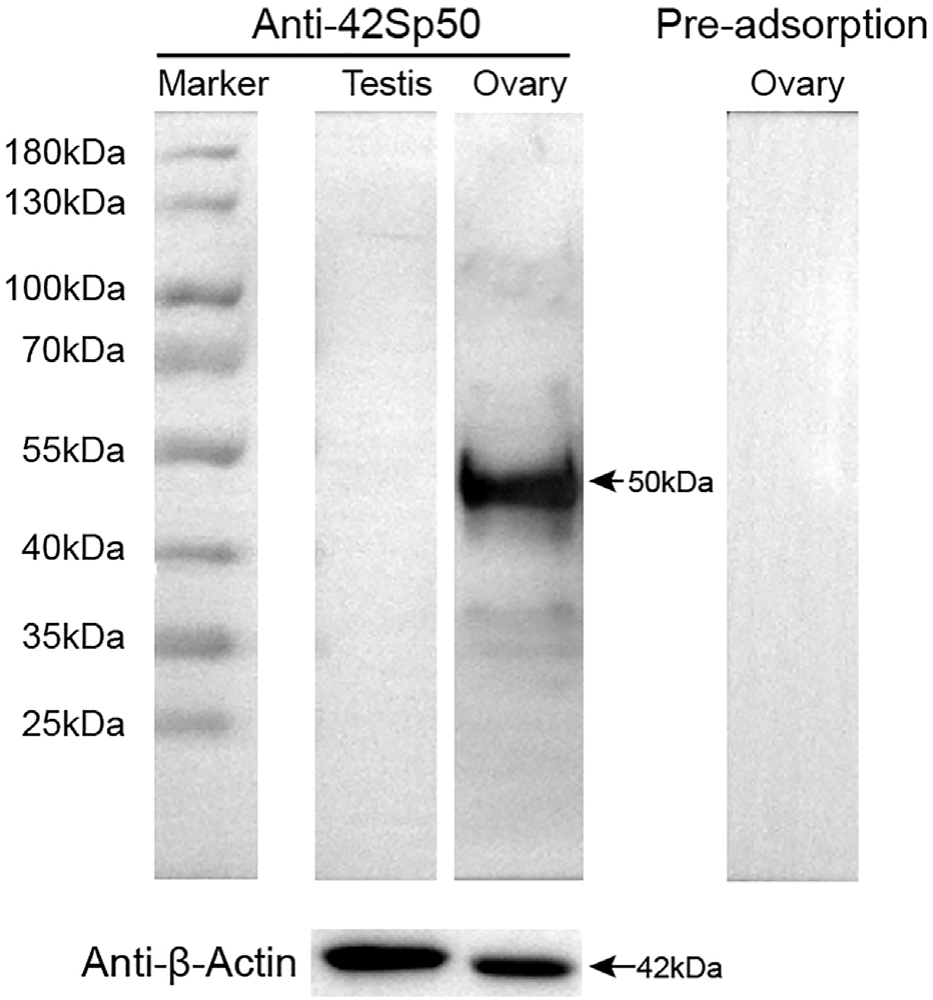
Specificity of anti-42Sp50 antibody analyzed by Western blotting. β*-Actin* abundance was examined as the loading control. The primary antibody which was preadsorbed with its respective recombination protein was used as negative controls.

In this study, spotted scat *42Sp50* polyclonal antibody was produced. The anti-*42Sp50* antibody detected a single 50.0 kDa band in the protein prepared from the spotted scat stage III ovary (Figure 5). The detected band size is similar to the calculated *42Sp50* protein size of 50.0 kDa. While *42Sp50* was almost undetectable in the testis. When the pre-adsorbed primary antibody was used, the *42Sp50* specific band of the ovary could not be detected (Figure 5).

### Expression of *42Sp50* in gonads at different developmental phases and E2 treated gonads by immunohistochemistry

**FIGURE 6:**
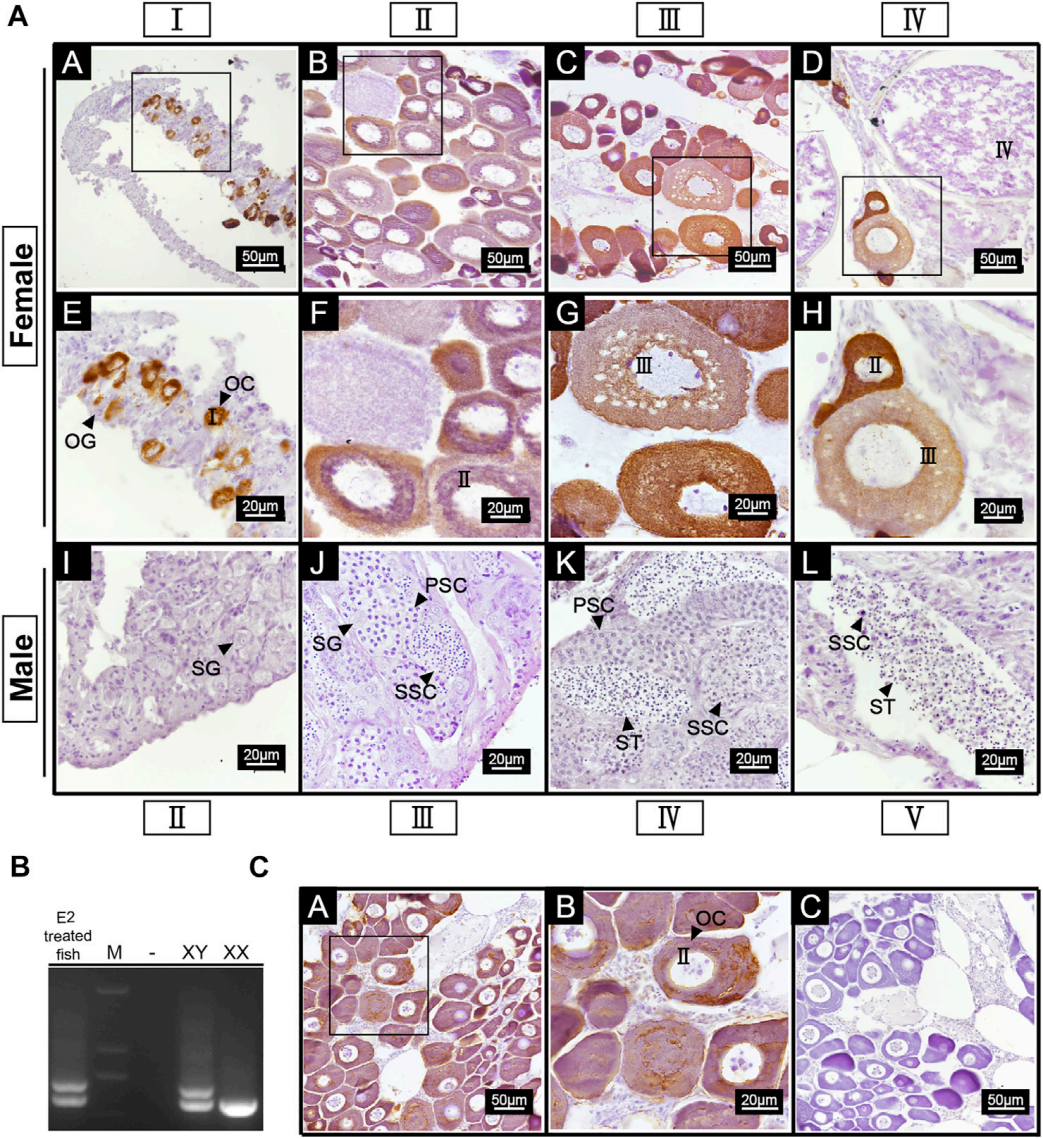
Cellular location of 42Sp50 in gonad of spotted scat by IHC. **(A)** A–H are ovaries at stage I, II, III and IV, respectively. E–H are the high magnifications of the boxed areas in A–D, respectively. I–L are testes at stage II, III, IV and V, respectively. **(B)** The identification of genetic sex of E2 treated fish. **(C)** A,B are the gonad of 2.5 years old E2 treated fish, B is the high magnification of the boxed area in **(A)** C is the negative control of E2 treated fish gonad. PBS was used instead of the primary antibody as the negative control.

IHC was carried out on ovaries of stage I-IV and the testis of stage II-V. The results showed positive signals in the ovaries of all stages. A strong anti-*42Sp50* positive signals were only observed in the cytoplasm of the oocytes at phase I and II, weak signals were observed in phase Ⅲ, and no signal was observed in the oocytes of phase IV. No positive signal was observed in the testes, indicating that low expression level of 42Sp50 in the testis (Figure 6A). In addition, gonads of 2.5 years old spotted scat were collected from the E2 group. The genetic sex of this fish is identified as XY (Figure 6B), but with ovarian features. The IHC results showed a positive signal in the cytoplasm of the oocyte, which was consistent with the result observed in the ovary of a wild-type female individual (Figure 6C). No positive signal was observed in the negative control (Figure 6C).

### Transcriptome analysis results

We obtained the mRNA expression data of *42Sp50* and its syntenic genes in five different teleosts (Supplementary Figure S2). The expression of *Myc*, *42Sp50,* and *Nfx1* was higher in females than in males, while *Fam49b* was higher in males than females. Different from them, the expressions of *Ankha* and *Asap1* differ among different species.

### Methylation levels of the *42Sp50* promoter

**FIGURE 7:**
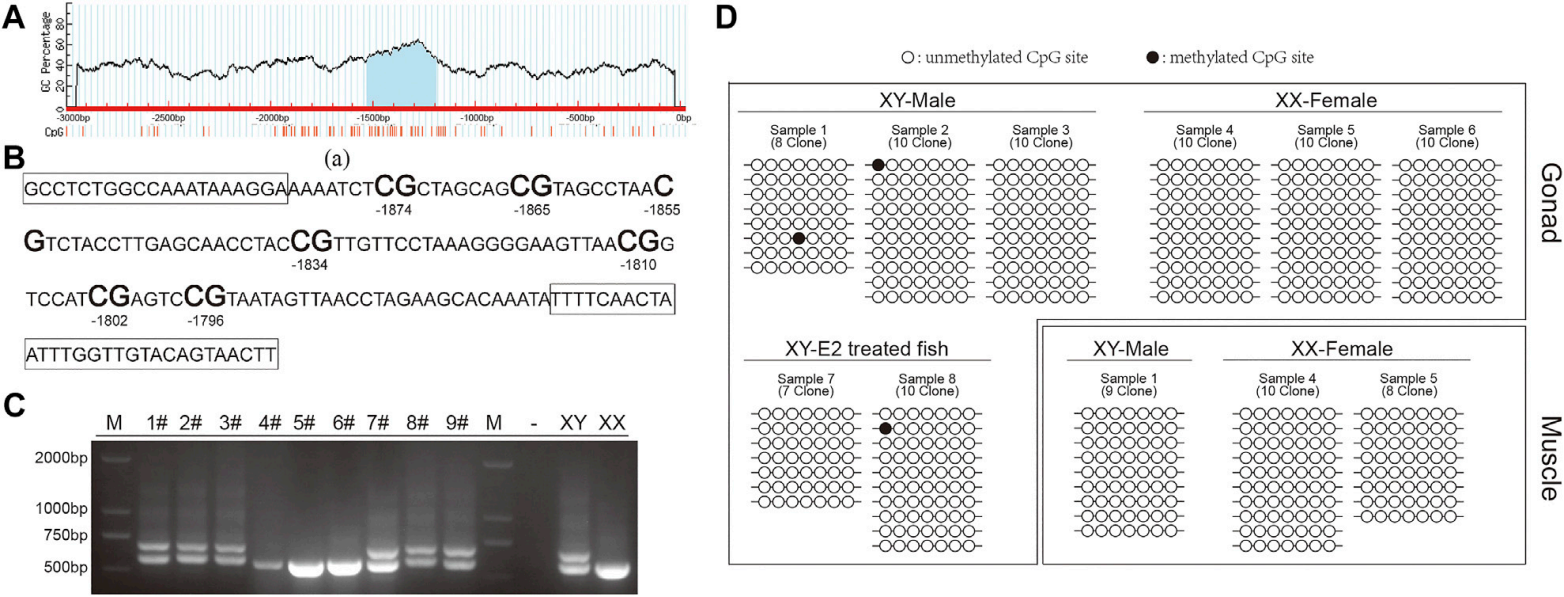
DNA methylation in the *42Sp50* promoter. **(A)** The prediction of CpG island in *42Sp50* promoter. **(B)** The fragment of *42Sp50* promoter selected in this study. The primers are marked with boxes. **(C)** The identification of genetic sex of E2 treated fish. **(D)** The site methylation level was analysed. Black solids and circles correspond to methylated and unmethylated sites, respectively.

According to the predictions, the *42Sp50* promoter has one CpG island, located between −1000 bp and −1500 bp (Figure 7A). The fragment selected for this experiment has 7 CG sites (Figure 7B). The genetic sex of nine fish treated with E2 were identified (Figure 7C), there are two bands in XY individuals and only one band in XX individuals. XY individuals are selected for the subsequent experiments. The comparison results show that the chosen site in this study has a lower degree of methylation whether in the gonads of XY-male, XX-female and XY-E2-treated fish and the muscles of XY-male and XX-female (Figure 7D).

### Luciferase assays of *42Sp50*

**FIGURE 8:**
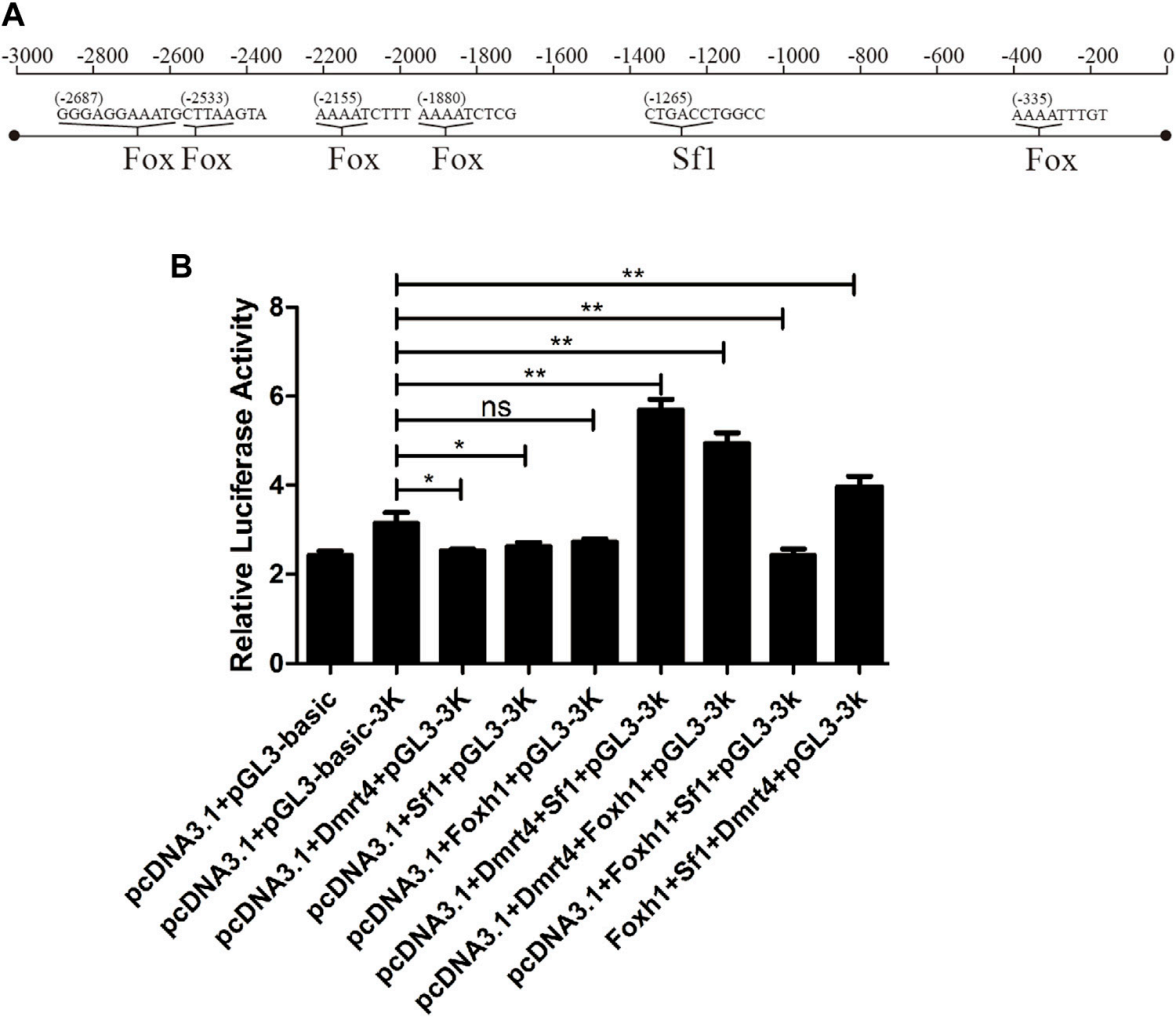
Regulation experiment of *42Sp50* promoter *in vitro*. **(A)** The binding sites of *42Sp50* promoter with *Sf1* and *Fox* gene family members predicted by PROMO. **(B)** Luciferase analysis to detect the regulatory effects of *Sf1*, *Foxh1*, and *Dmrt4* and their combinations on the promoter of *42Sp50*.

Figure 8A shows the binding sites of *42sp50* promoter with *Sf1* and *Fox* gene family members predicted by PROMO. The result of luciferase assays showed that *42sp50* was not activated when *Foxh1*, *Sf1,* and *Dmrt4* were alone, respectively (Figure 8B). In the combination of two transcription factors, *Sf1* or *Foxh1* could activate the transcription of *42Sp50* when they were present together with *Dmrt4*. Among the combinations of three transcription factors, the combination of *Dmrt4*, *Foxh1,* and *Sf1* promoted the transcription of *42Sp50*. Other combinations had no effect on the transcription of *42Sp50*.

## Discussion

*42Sp50* is a member of *eEF1A* family, which is lost during evolution in some vertebrates, including mammals and birds. Expression and functional analysis of *42Sp50* showed it is highly expressed in the ovary and necessary for oocyte development. While its transcriptional regulation is rarely reported. The partial cDNA of spotted scat *42Sp50* was cloned in the present study. Genome-wide searching of *42Sp50* in vertebrates confirmed that it was lost in some tetrapod groups. Spotted scat *42Sp50* showed a similar expression pattern with other fish, indicating its conserved role of it in ovary development. DNA methylation and *in vitro* promoter analysis of *42Sp50* were carried out to reveal the possible mechanisms for its ovary specific expression in spotted scat.

### *42Sp50* was evolutionarily lost in some tetrapod species

Gene duplication usually happens during evolution and is important for acquiring novel genes promoting the evolution of organisms (Magadum et al., 2013). There are three rounds of genome duplication during the evolution of vertebrates, named 1, 2, and 3R. 1R happened before and 2R after Cyclostomata formation (Steinke et al., 2006). While the 3R, or fish-specific genome duplication (FSGD), specifically occurred in the ancestor of teleosts, and most of the duplicated genes were secondarily lost (Hoegg et al., 2004; Meyer and Van de Peer, 2005). Herein we identified *42Sp50,* which is located in a conserved gene cluster in fish and some tetrapods. Meanwhile, *42Sp50* could not be found in lamprey, indicating that *42Sp50* appears after the 2R before 3R in the vertebrates. Only one *42Sp50* was found in the analyzed teleosts indicating that this gene did not duplicate during the 3R or its duplicates were secondarily lost after 3R. As *42Sp50* is a reproduction-related gene, its disappearance in some tetrapods might be caused by the change of reproduction strategy, which makes this gene redundant and thereafter lost. There are a lot of reproduction-related genes lost during species diversification and evolution. *Dmrt6* exists in Nile tilapia and stickleback, while it is not found in zebrafish, medaka, spot gar, and mammals (Zhang et al., 2014). Similar with *42Sp50*, *Gsdf* exists in most fish, including spotted scat and some tetrapods (Hsu and Chung, 2021). Interestingly, protein interaction analysis showed that Gsdf could interact with eEF1a3, which is homologous to *42Sp50* in medaka (Zhang et al., 2021). It is interesting to test if *42Sp50* could also interact with Gsdf in fish. If *42Sp50* and *Gsdf* are functionally related and evolutionary lost in similar species, we can propose that functionally related genes will be lost together during evolution. Genes like *42Sp50* and *Gsdf* are very good models for evolutionary gene analysis. They could increase our knowledge of the rule of gene loss in vertebrates.

### The conservation of *42Sp50* expression

In the present study, RT-PCR, qPCR, Western blot and IHC results showed that *42Sp50* was predominantly and highly expressed in the ovaries of spotted scat, showing a sex-dimorphic expression pattern. This expression pattern is identical to that observed in Nile tilapia (Wei, 2011; Chen, 2018). Consistently, the transcriptome of gonads in several unrelated fish confirmed that *42Sp50* also expressed much higher in ovary than that of testis. In *X. laevis*, *42Sp50* was expressed abundantly in previtellogenic oocytes (Viel et al., 1991). Similarly, *42Sp50* was highly expressed in the phase I and II oocytes in spotted scat and Nile tilapia indicating that *42Sp50* might be critical for early oocytes development (Chen, 2018). In XX *42Sp50* ^−/−^ Nile tilapia, the development of the oocytes was blocked at the early stages, confirming that *42Sp50* is necessary for ovary development (Chen, 2018). However, the detailed mechanism of *42Sp50* in oocyte development remains unclear. *42Sp50* might act as its family member`s classical function as protein synthesis factor to transfer aminoacyl tRNA to the ribosomes during elongation. *42Sp50* is present in the 42 S particles and binds with tRNA transcribed in oocytes at the early stage of vitellogenesis, thus *42Sp50* might be important for long-term storage of aminoacyl tRNA in early oocyte development stages (Viel et al., 1987). It is well documented that the location and transcription of the RNA in the oocytes is delicately regulated, and the maternal RNA is critical for embryos development (Miao et al., 2017). The expression of *42Sp50* is deceased in the mature oocyte and lowly expressed in the embryo, indicating that it did not act as maternal protein (Supplementary Figure S3). Alternatively, *42Sp50* might participate in the protection, location and transcription regulation of RNA in the oocytes at the early stages. In future studies, the detailed mechanism of *42Sp50* in oocyte development should be addressed.

Considering the special expression pattern of *42Sp50*, it could be taken as the oocytes marker gene in fish (Kinoshita et al., 2009; Chen, 2018). Recently, some gonads of XY male spotted scat individuals were found to develop abnormally with occurrence of primary oocytes (PO) in testes and was referred to as ectopic primary oocytes (Ecto-PO), whereas *42Sp50* was found to be expressed in the Ecto-PO (Mustapha et al., 2022). It is still unknown why and how the appearance of the Ecto-PO, and whether the fertility of the individuals containing Ecto-PO would be influenced. *42Sp50* is a good marker gene which could be used for tracing the Ecto-PO in the intersex male fish. There will be a broad application of *42Sp50* antibody prepared in the present study in future artificial reproduction related studies in spotted scat.

### The reason for the ovary special expression of *42Sp50*

Numerous genes show sexually biased expression patterns in gonads, and the detailed transcription regulation mechanisms for those genes are not widely studied. Through RNA-Seq, a total of 32,122 differentially expressed genes (DEGs) were identified in the gonads of spotted scat, of which 11,156 were highly expressed in the testis and 20,966 were highly expressed in the ovary (He et al., 2019). Some gene clusters containing many genes showed similar expression patterns, e.g., the genes located near *Gsdf* were highly expressed in fish ovaries (Gautier et al., 2011). However, the adjacent genes of *42Sp50* are not consistently highly expressed in ovaries, so *42Sp50* is not located within a gene cluster which consists of several ovary biased expressed genes. DNA Methylation is a chemical modification of DNA that can regulate gene expression. DNA methylation may be one reason for genes’ sexual dimorpic expression pattern. In European sea bass (*Dicentrarchus labrax*), the testicular DNA methylation level of *Cyp19a* promoter is higher than that of ovary, resulting in the higher expression of *Cyp19a* in ovaries than that of testes (Navarro-Martín et al., 2011). In barramundi (*Lates calcarifer*), the promoter DNA methylation degree of sex biased genes *Dmrt1* and *NR5A2* was higher in females, and the gene expression was also negatively correlated with their promoter DNA methylation level (Domingos et al., 2018). In this study, the DNA methylation levels of *42Sp50* promoter were all very low in testes, ovaries and muscles. Thus it is speculated that the expression of *42Sp50* does not depend on the DNA methylation regulation of its promoter. Transcription factors can regulate gene expression by interacting with transcription factor binding sites of the target gene promoter. Sexual biased expression of genes can be attributed to their transcription factors. Herein, *42Sp50* was found to be regulated by *Sf1* or *Foxh1* in the presence of *Dmrt4 in vitro* in spotted scat. *Dmrt4* seems to be essential for *42Sp50* transcription*.* In blue tilapia (*Oreochromis aurea*), IHC analysis showed that strong Dmrt4 signals were observed in the cytoplasm of early previtellogenic oocytes and male germ cells at stages from A-type spermatogonia to primary spermatocytes (Cao et al., 2007). In spotted scat, *Dmrt4* is highly expressed in the ovaries and testes (Jiang et al., 2019). While *Dmrt4* is relatively expressed higher in testis than that of ovary in spotted scat. This seems to conflict with our results that *42Sp50* was only weakly expressed in the testis. To interpret this, we can deduce that *Dmrt4* alone can not activate *42Sp50* expression in the testis, and this speculation is consistent with the *in vitro* analysis. Alternatively, some transcription factors could restrict the *42Sp50* expression in the testis in spotted scat. In Nile tilapia, IHC analysis showed that Sf1 was expressed in the oogonia, interstitial cells, granulosa cells, theca cells of the ovary, and spermatogonia Leydig cells of the testis at 90 and 180 days after hatching (Xie et al., 2016). The cellular location of *42Sp50* and Sf1 are not overlapped, indicating that our *in vitro* results might be artifact. While we still can not exclude that Sf1 might be expressed in the oocyte at phase I and II at lower level, which was undetectable by IHC. In Nile tilapia, ISH showed that *Foxh1* has a sexually dimorphic expression pattern in gonads, with extremely high expression in phase I and II oocytes (Yuan et al., 2014). Herein, tissue distribution analysis showed that *Foxh1* was specifically expressed in the ovary (Supplementary Figure S4). So female-specific *Foxh1* might be the reason for the higher expression of its target gene *42Sp50* in the ovary. In addition, the *Foxh1* ^−/−^ XX Nile tilapia also showed oogenesis arrest similar to that of *42Sp50* knockout XX fish (Tao et al., 2019). Foxh1 and *42Sp50* might also be functionally related in fish.

## Conclusion

Herein, *42Sp50* was cloned from the gonads of spotted scat. *42Sp50* gene was identified in some reptile species, while it was evolutionarily lost in all birds and mammals. *42Sp50* was expressed higher in ovaries than that of testis in spotted scat and mainly expressed in the cytoplasm of phase I and II oocytes indicating that it is critical for ovarian development in spotted scat. The expression of *42Sp50* might not be regulated by the DNA methylation modification of its promoter. The luciferase reporter assay demonstrated that *Dmrt4* activated *42Sp50* expression in the presence of *Sf1* or *Foxh1* in spotted scat. This is the first report of the transcription regulation study of *42Sp50*. *42Sp50* is proved to be a good oocytes marker gene in fish.

## Data Availability

The datasets presented in this study can be found in online repositories. The names of the repository/repositories and accession number(s) can be found below: https://www.ncbi.nlm.nih.gov/, MT774144.

## References

[B1] Barry T. P. Castanos M. T. Macahilig M. P. S. C. Fast A. W. (1993). Gonadal maturatio and spawning induction in female spotted scat (*Scatophagus argus*). J. Aquac. Trop. 8, 121–130. 10.1016/j.anireprosci.2020.106273

[B2] Brands J. H. Maassen J. A. Van Hemert F. J. Amons R. Möller W. (1986). The primary structure of the alpha subunit of human elongation factor 1. Structural aspects of guanine-nucleotide-binding sites. Eur. J. Biochem. 155 (1), 167–171. 10.1111/j.1432-1033.1986.tb09472.x 3512269

[B3] Browne G. J. Proud C. G. (2002). Regulation of peptide-chain elongation in mammalian cells. Eur. J. Biochem. 269 (22), 5360–5368. 10.1046/j.1432-1033.2002.03290.x 12423334

[B4] Cao J. Cao Z. Wu T. (2007). Generation of antibodies against DMRT1 and DMRT4 of *Oreochromis aurea* and analysis of their expression profile in *Oreochromis aurea* tissues. J. Genet. Genomics 34 (6), 497–509. 10.1016/S1673-8527(07)60055-1 17601609

[B5] Chambers D. M. Peters J. Abbott C. M. (1998). The lethal mutation of the mouse wasted (*wst*) is a deletion that abolishes expression of a tissue-specific isoform of translation elongation factor 1alpha, encoded by the *Eef1a2* gene. Proc. Natl. Acad. Sci. U. S. A. 95 (8), 4463–4468. 10.1073/pnas.95.8.4463 9539760 PMC22512

[B6] Chen J. L. (2018). The role of elongation factor eEF1A1b and 42Sp50 in gametogenesis in Nile tilapia. Doctor Thesis. Chongqing, China: Southwest University, 1–125. Available at: https://kns.cnki.net/kcms/detail/detail.aspx? (Supervisor: Wang D.S).

[B7] Cui D. Liu Z. W. Liu N. X. Zhang Y. Y. Zhang J. B. (2013). Histological study on the gonadal development of *Scatophagus argus* . J. Fish. China 7 (05), 696. 10.3724/SP.J.1231.2013.38442

[B8] Deschamps S. Morales J. Mazabraud A. Le Maire M. Denis H. Brown D. D. (1991). Two forms of elongation factor 1 alpha (EF-1 alpha O and 42Sp50), present in oocytes, but absent in somatic cells of *Xenopus laevis* . J. Cell Biol. 114 (6), 1109–1111. 10.1083/jcb.114.6.1109 1894690 PMC2289126

[B9] Djumagulov M. Demeshkina N. Jenner L. Rozov A. Yusupov M. Yusupova G. (2021). Accuracy mechanism of eukaryotic ribosome translocation. Nature 600 (7889), 543–546. 10.1038/s41586-021-04131-9 34853469 PMC8674143

[B10] Domingos J. A. Budd A. M. Banh Q. Q. Goldsbury J. A. Zenger K. R. Jerry D. R. (2018). Sex-specific *dmrt1* and *cyp19a1* methylation and alternative splicing in gonads of the protandrous hermaphrodite barramundi. PLoS One 13 (9), e0204182. 10.1371/journal.pone.0204182 30226860 PMC6143260

[B11] Dong Z. D. Li X. Y. Yao Z. B. Wang C. Guo Y. S. Wang Q. (2021). *Oryzias curvinotus* in Sanya does not contain the male sex-determining gene *dmy* . Animals. 11 (5), 1327. 10.3390/ani11051327 34066583 PMC8148570

[B12] Gautier A. Le Gac F. Lareyre J. J. (2011). The *gsdf* gene locus harbors evolutionary conserved and clustered genes preferentially expressed in fish previtellogenic oocytes. Gene 472 (1-2), 7–17. 10.1016/j.gene.2010.10.014 21047546

[B13] He F. X. Jiang D. N. Huang Y. Q. Mustapha U. F. Yang W. Cui X. F. (2019). Comparative transcriptome analysis of male and female gonads reveals sex-biased genes in spotted scat (*Scatophagus argus*). Fish. Physiol. Biochem. 45 (6), 1963–1980. 10.1007/s10695-019-00693-8 31399918

[B14] Hoegg S. Brinkmann H. Taylor J. S. Meyer A. (2004). Phylogenetic timing of the fish-specific genome duplication correlates with the diversification of teleost fish. J. Mol. Evol. 59 (2), 190–203. 10.1007/s00239-004-2613-z 15486693

[B15] Holmseth S. Zhou Y. Follin-Arbelet V. V. Lehre K. P. Bergles D. E. Danbolt N. C. (2012). Specificity controls for immunocytochemistry: the antigen preadsorption test can lead to inaccurate assessment of antibody specificity. J. Histochem. Cytochem. 60 (3), 174–187. 10.1369/0022155411434828 22215633 PMC3351124

[B16] Hsu C. W. Chung B. C. (2021). Evolution, expression, and function of gonadal somatic cell-derived factor. Front. Cell Dev. Biol. 9, 684352. 10.3389/fcell.2021.684352 34307362 PMC8292791

[B17] Huang Y. Huang Y. Q. Deng Q. M. Mustapha U. F. Peng Y. X. Li G. L. (2022). A rapid method for genetic sex identification in the spotted scat (*Scatophagus argus*). J. Fish. Sci. China 29 (04), 515–524. 10.12264/JFSC2021-0359

[B18] Idigo N. J. Soares D. C. Abbott C. M. (2020). Translation elongation factor 1A2 is encoded by one of four closely related *eef1a* genes and is dispensable for survival in zebrafish. Biosci. Rep. 40 (1), BSR20194191. 10.1042/BSR20194191 31950975 PMC6997148

[B19] Infante C. Asensio E. Cañavate J. P. Manchado M. (2008). Molecular characterization and expression analysis of five different elongation factor 1 alpha genes in the flatfish Senegalese sole (*Solea senegalensis* kaup): differential gene expression and thyroid hormones dependence during metamorphosis. BMC Mol. Biol. 30 (9), 19. 10.1186/1471-2199-9-19 PMC2270864 18234081

[B20] Janssen G. M. Möller W. (1988). Kinetic studies on the role of elongation factors 1 beta and 1 gamma in protein synthesis. J. Biol. Chem. 263 (4), 1773–1778. 10.1016/s0021-9258(19)77943-5 3338993

[B21] Jiang D. N. Peng Y. X. Mustapha U. K. Gu H. T. Deng S. P. Chen H. P. (2019). Molecular cloning and expression profile of *Dmrt4* in spotted scat (*Scatophagus argus*). J. Guangdong Ocean. Univ. 39 (01), 7–13. 10.3969/j.issn.1673-9159.2019.01.002

[B22] Kahns S. Lund A. Kristensen P. Knudsen C. R. Clark B. F. Cavallius J. (1998). The elongation factor 1 A-2 isoform from rabbit: cloning of the cDNA and characterization of the protein. Nucleic Acids Res. 26 (8), 1884–1890. 10.1093/nar/26.8.1884 9518480 PMC147499

[B23] Kinoshita M. Okamoto G. Hirata T. Shinomiya A. Kobayashi T. Kubo Y. (2009). Transgenic medaka enables easy oocytes detection in live fish. Mol. Reprod. Dev. 76 (2), 202–207. 10.1002/mrd.20942 18543284

[B24] Knudsen S. M. Frydenberg J. Clark B. F. Leffers H. (1993). Tissue-dependent variation in the expression of elongation factor-1 alpha isoforms:isolation and characterisation of a cDNA encoding a novel variant of human elongation-factor 1 alpha. Eur. J. Biochem. 215 (3), 549–554. 10.1111/j.1432-1033.1993.tb18064.x 8354261

[B25] Lee S. Ann D. K. Wang E. (1994). Cloning of human and mouse brain cDNAs coding for S1, the second member of the mammalian elongation factor-1 alpha gene family: analysis of a possible evolutionary pathway. Biochem. Biophys. Res. Commun. 203 (3), 1371–1377. 10.1006/bbrc.1994.2336 7945283

[B26] Lin X. H. Zhou D. Y. Zhang X. M. Li G. L. Zhang Y. L. Huang C. L. (2021). A first insight into the gonad transcriptome of Hong Kong catfish (*Clarias fuscus*). Animals. 11 (4), 1131. 10.3390/ani11041131 33920938 PMC8071282

[B27] Livak K. J. Schmittgen T. D. (2001). Analysis of relative gene expression data using real-time quantitative PCR and the 2^−ΔΔCt^ Method. Methods 25 (4), 402–408. 10.1006/meth.2001.1262 11846609

[B28] Magadum S. Banerjee U. Murugan P. Gangapur D. Ravikesavan R. (2013). Gene duplication as a major force in evolution. J. Genet. 92 (1), 155–161. 10.1007/s12041-013-0212-8 23640422

[B29] Mandal B. Kailasam M. Bera A. Sukumaran K. Hussain T. Biswas G. (2021). Standardization of oocyte size during artificial fertilization and optimization of stocking density during indoor larval and outdoor nursery rearing of captive spotted scat (*Scatophagus argus*) for a viable juvenile production system. Aquaculture 534, 736262. 10.1016/j.aquaculture.2020.736262

[B30] Meyer A. Van de Peer Y. (2005). From 2R to 3R: evidence for a fish-specific genome duplication (FSGD). Bioessays 27, 937–945. 10.1002/bies.20293 16108068

[B31] Miao L. Y. Yuan Y. Cheng F. Fang J. J. Zhou F. Ma W. R. (2017). Translation repression by maternal RNA binding protein Zar1 is essential for early oogenesis in zebrafish. Development 144 (1), 128–138. 10.1242/dev.144642 27913641

[B32] Mustapha U. F. Huang Y. Huang Y. Q. Assan D. Shi H. J. Jiang M. Y. (2021). Gonadal development and molecular analysis revealed the critical window for sex differentiation, and E2 reversibility of XY-male spotted scat, *Scatophagus argus* . Aquaculture 544, 737147. 10.1016/j.aquaculture.2021.737147

[B33] Mustapha U. F. Zhi F. Huan Y. Q. Assan D. Li G. L. Jiang D. N. (2022). First account of a transient intersex in spotted scat, *Scatophagus argus*: a marine gonochoristic fish. Fish. Physiol. Biochem. 10.1007/s10695-022-01097-x 35804212

[B34] Navarro-Martin L. Vinas J. Ribas L. Diaz N. Gutierrez A. Di Croce L. (2011). DNA methylation of the gonadal aromatase (*cyp19a*) promoter is involved in temperature-dependent sex ratio shifts in the European sea bass. PLoS Genet. 7 (12), e1002447. 10.1371/journal.pgen.1002447 22242011 PMC3248465

[B35] Ru X. Y. Shi H. J. Wang T. Liu Q. Q. Jiang D. N. Peng Y. H. (2020). Effects of 17β-Estradiol on growth-related genes expression in female and male spotted scat (*Scatophagus argus*). Comp. Biochem. Physiol. B Biochem. Mol. Biol. 250, 110492. 10.1016/j.cbpb.2020.110492 32889045

[B36] Steinke D. Hoegg S. Brinkmann H. Meyer A. (2006). Three rounds (1R/2R/3R) of genome duplications and the evolution of the glycolytic pathway in vertebrates. BMC Biol. 4, 16. 10.1186/1741-7007-4-16 16756667 PMC1508162

[B37] Tao W. J. Shi H. J. Yang J. Diakite H. D. Kocher T. D. Wang D. S. (2019). Homozygous mutation of *foxh1* arrests oogenesis causing infertility in female Nile tilapia. Biol. Reprod. 102 (3), 758–769. 10.1093/biolre/ioz225 31837141

[B50] Tao W. Yuan J. Zhou L. Sun L. Sun Y. Yang S. (2013). Characterization of Gonadal transcriptomes from Nile tilapia (*Oreochromis niloticus*) reveals differentially expressed genes. PLoS One 8, 63604. 10.1371/journal.pone.0063604 PMC3643912 23658843

[B38] Tian C. X. Li Z. Y. Dong Z. D. Huang Y. Du T. Chen H. P. (2019). Transcriptome analysis of male and female mature gonads of silver sillago (*Sillago sihama*). Genes 10 (2), 129. 10.3390/genes10020129 PMC6409516 30754713

[B39] Viel A. Djé M. K. Mazabraud A. Denis H. le Maire M. (1987). Thesaurin a, the major protein of *Xenopus laevis* previtellogenic oocytes, present in the 42 S particles, is homologous to elongation factor EF-1 alpha. FEBS Lett. 223 (2), 232–236. 10.1016/0014-5793(87)80295-8 3666148

[B40] Viel A. Le Maire M. Philippe H. Morales J. Mazabraud A. Denis H. (1991). Structural and functional properties of thesaurin a (42Sp50), the major protein of the 42 S particles present in *Xenopus laevis* previtellogenic oocytes. J. Biol. Chem. 266 (16), 10392–10399. 10.1016/S0021-9258(18)99238-0 2037589

[B41] Wang H. Parent M. Morais R. (1994). Cloning and characterization of a cDNA encoding elongation factor 1 alpha from chicken cells devoid of mitochondrial DNA. Gene 140 (2), 155–161. 10.1016/0378-1119(94)90539-8 8144022

[B42] Wang D. S. Kobayashi T. Zhou L. Y. Paul-Prasanth B. Ijiri S. Sakai F. (2007). Foxl2 up-regulates aromatase gene transcription in a female-specific manner by binding to the promoter as well as interacting with ad4 binding protein/steroidogenic factor 1. Mol. Endocrinol. 21 (3), 712–725. 10.1210/me.2006-0248 17192407

[B43] Wegnez M. Denis H. (1979). Biochemical research on oogenesis. Transfer RNA is fully charged in the 42-S storage particles of *Xenopus laevis* oocytes. Eur. J. Biochem. 98 (1), 67–75. 10.1111/j.1432-1033.1979.tb13161.x 467449

[B44] Wei Y. Y. (2011). Molecular cloning and expression of translation elongation fascors (eEF1A) in the Nile tilapia. Master Thesis. Chongqing, China: Southwest University, 1–51. Available at: https://kns.cnki.net/kcms/detail/detail.aspx? (Supervisor: Wang D. S).

[B45] Xie Q. P. He X. Sui Y. N. Chen L. L. Sun L. N. Wang D. S. (2016). Haploinsufficiency of SF-1 causes female to male sex reversal in nile Tilapia, *Oreochromis niloticus* . Endocrinology 157 (6), 2500–2514. 10.1210/en.2015-2049 27046435

[B46] Yoshikawa A. Takano-ohmuro H. Masaki T. (1984). Increase in the amount of elongation factor 2 in chicken muscular dystrophy. Muscle Nerve 7 (9), 733–740. 10.1002/mus.880070907 6543921

[B47] Yuan J. Tao W. J. Cheng Y. Y. Huang B. F. Wang D. S. (2014). Genome-wide identification, phylogeny, and gonadal expression of *fox* genes in Nile tilapia, *Oreochromis niloticus* . Fish Physiol. Biochem. 40 (4), 1239–1252. 10.1007/s10695-014-9919-6 24526262

[B48] Zhang X. B. Wang H. Li M. H. Cheng Y. Y. Jiang D. N. Sun L. N. (2014). Isolation of doublesex- and *mab-3-*related transcription factor 6 and its involvement in spermatogenesis in tilapia. Biol. Reprod. 91 (6), 136. 10.1095/biolreprod.114.121418 25320148

[B49] Zhang X. T. Chang Y. Y. Zhai W. Y. Qian F. Zhang Y. Q. Xu S. M. (2021). A potential role for the gsdf-eEF1α complex in inhibiting germ cell proliferation: A protein-interaction analysis in medaka (*Oryzias latipes*) from a proteomics perspective. Mol. Cell Proteomics 20, 100023. 10.1074/mcp.RA120.002306 33293461 PMC7950199

