# Supplementary material for: Expression and regulation of 42Sp50 in spotted scat (Scatophagus argus)
Source: Front Genet. 2022 Aug 11;13:964150. doi: 10.3389/fgene.2022.964150 (PMC9403048; doi:10.3389/fgene.2022.964150)
Supplement: Supplementary file 1 [file DataSheet1.docx]

Supplementary Material


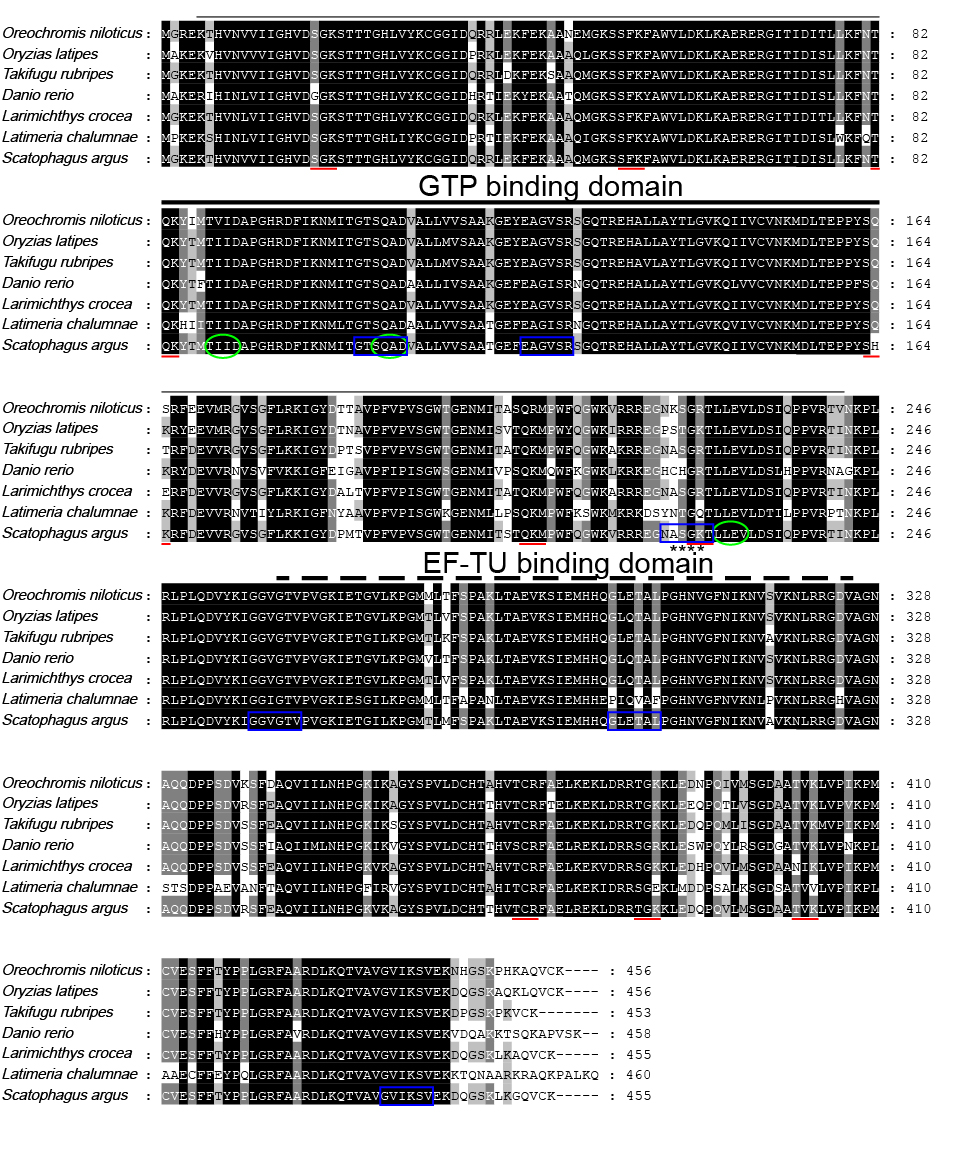


**Figure S1.** Alignment of the deduced amino acid sequence of spotted scat (*Scatophagus argus*) 42Sp50 with those of other teleosts. “-” , no amino acid; Black solid line, the GTP domain; Dotted line, EF-TU domain;“*”, N-glycosylation site; Red solid line, Protein kinase C phosphorylation site; Green circle, Casein kinase II phosphorylation site; Blue box, N-myristoylation sites.


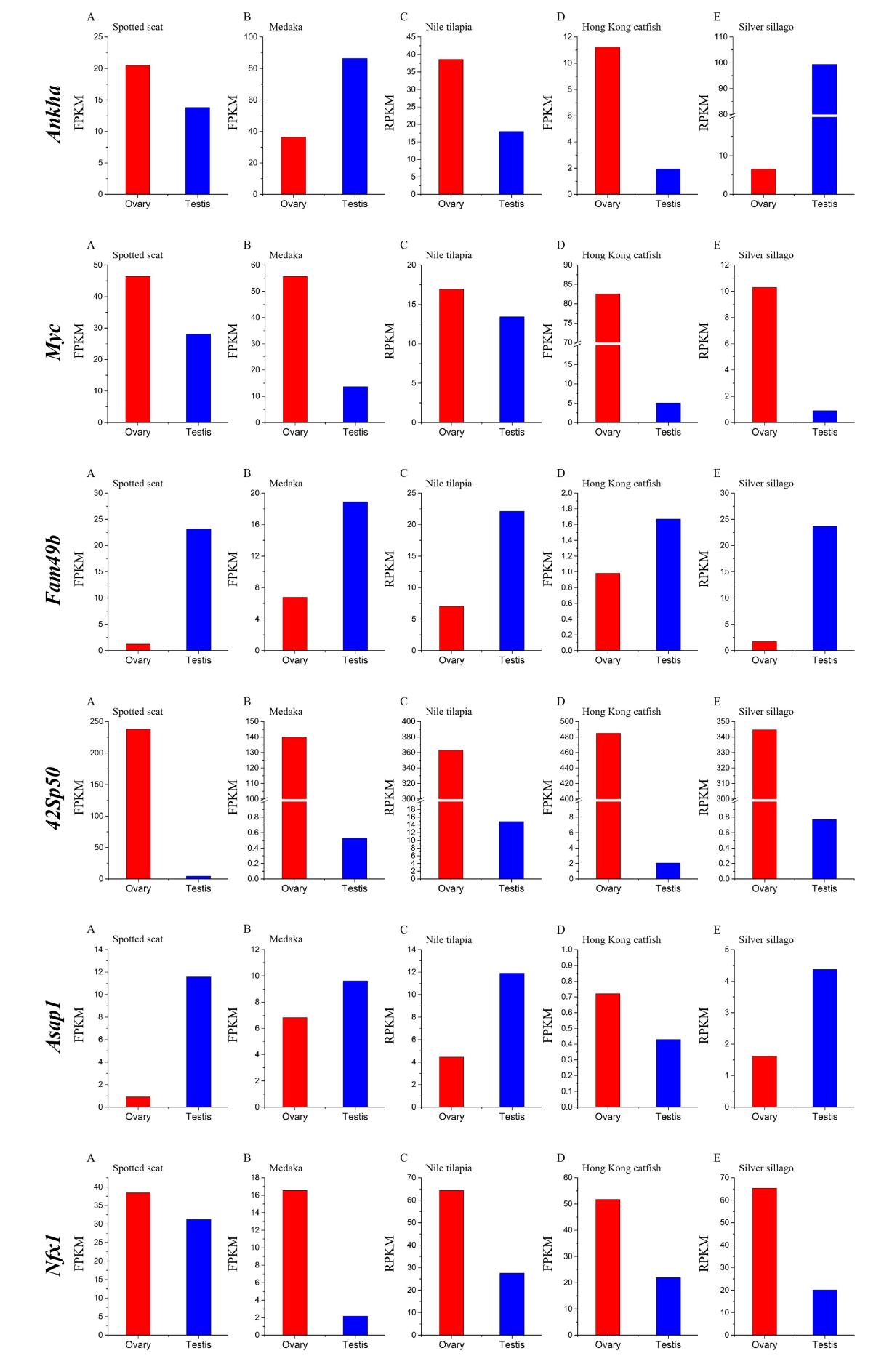


**Figure S2.** Transcriptome expression of *42Sp50* and its syntenic genes in five different teleosts.


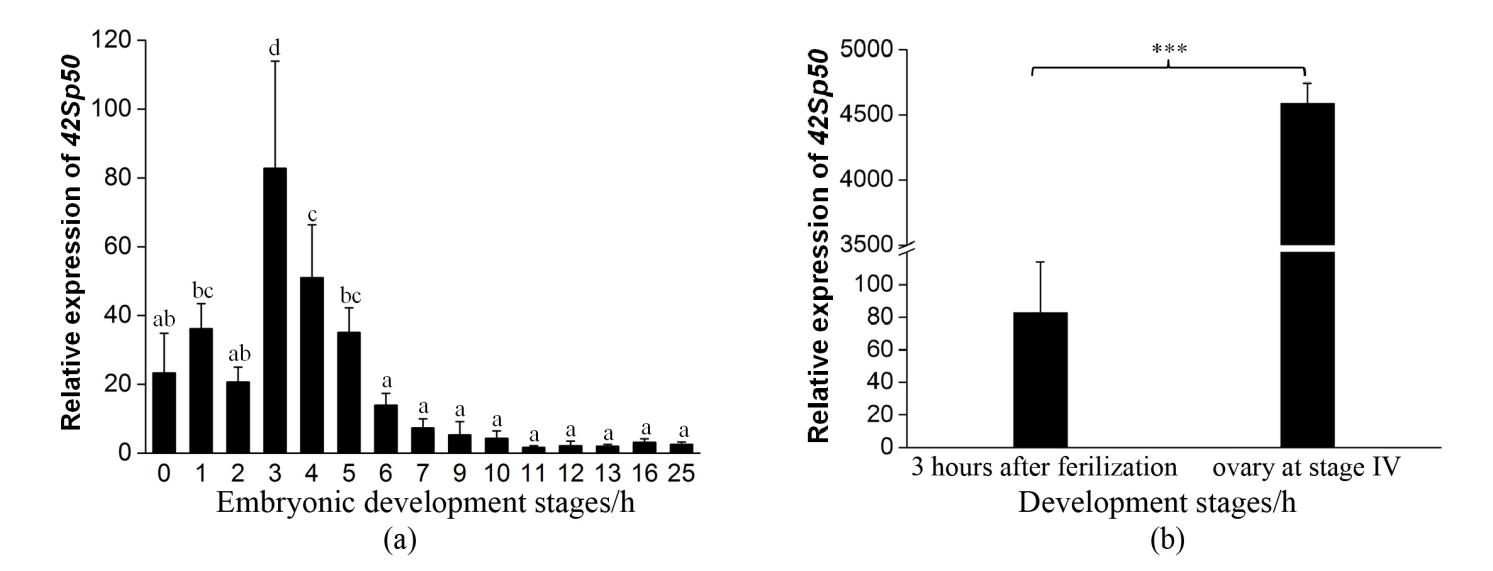


**Figure S3.** The expression of *42Sp50* in different stages of embryonic development. (a) The expression of *42Sp50* in different stages of embryonic development. (b) Comparison of the expression levels of *42Sp50* in 3 hours after fertilization and ovary at stage Ⅳ. Significant differences at p < 0.05 were labeled with different letters.


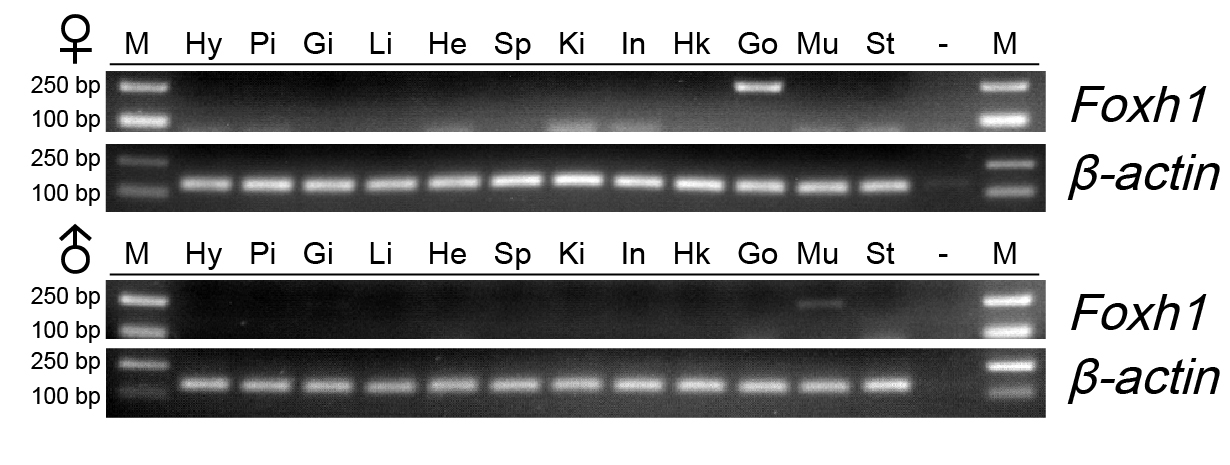


**Figure S4.** Tissue distribution of *Foxh1* in spotted scat. Hy, hypothalamus; P, pituitary; Gi, gill; L, liver; He, heart; Sp, spleen; K, kidney; St, stomach; I, intestines; Go, gonad; Mu, muscle; "−", negative control; Marker, DS™2000.

**Supplementary Table S1.** Primer sequences used in this study.

| **Gene** | **Primer name** | **Primer sequence (5′-3′)** | **Purpose** |
| --- | --- | --- | --- |
| *42Sp50* | *42Sp50*-orf-F | CCATAGTGGCAGGTAAAATCC | clone |
|  | *42Sp50*-orf-R | TGTGTCACAACACAAATTGCAT | clone |
|  | *42Sp50*-td-F | GACCAGCCGCAGGTACTGATGT | qRT-PCR |
|  | *42Sp50*-td-R | GAGAAGGATCAAGGGTCAAAACTC | qRT-PCR |
|  | *42Sp50*-JJH-F | GTTTTTGGTTAAATAAAGGAA | DNA bisulfite treatment |
|  | *42Sp50-*JJH-R | AAATTACTATACAACCAAATTAATTAAAAA | DNA bisulfite treatment |
|  | *42Sp50-p-*3k-F | CGACGCGTTGCCAAAGGGGACAAGTCAAATAAC | luciferase assays |
|  | *42Sp50*-p-R | GGACTCGAGAGTTGAAGGCAGATTTTGCTAACCAG | luciferase assays |
| *β-actin* | *Actin*-F | GAGAGGTTCCGTTGCCCAGAG | qRT-PCR |
|  | *Actin*-R | CAGACAGCACAGTGTTGGCGT | qRT-PCR |
| *Dmrt1* | *Dmrt1*-Marker-4-F4 | TCAGAGCACAATTGTTAGGCAAAGTGAAC | gender identification |
|  | *Dmrt1*-Marker-4-R4 | TCGCTACTTTCACCAATACAGCATGA | gender identification |

**Supplementary Table 2.** The accession numbers of 42Sp50 and Eef1b2 in selected vertebrate species used in phylogenetic tree.

| **Species** | **Abbreviation** | **Accession ID** |
| --- | --- | --- |
| *Trachemys scripta elegans* | *T. scripta* | XP_034619513.1 |
| *Chelonoidis abingdonii* | *C. abingdonii* | XP_032618809.1 |
| *Gopherus evgoodei* | *G. evgoodei* | XP_030405473.1 |
| *Terrapene carolina triunguis* | *T. carolina triunguis* | XP_026515721.1 |
| *Chrysemys picta* | *C. picta* | XP_005288884.3 |
| *Chelonia mydas* | *C. mydas* | XP_037749228.1 |
| *Dermochelys coriacea* | *D. coriacea* | XP_038246522.2 |
| *Pelodiscus sinensis* | *P. sinensis* | XP_025040663.1 |
| *Alligator sinensis* | *A. sinensis* | XP_025061199.1 |
| *Crocodylus porosus* | *C. porosus* | XP_019408390.1 |
| *Gavialis gangeticus* | *G. gangeticus* | XP_019375129.1 |
| *Alligator mississippiensis* | *A. mississippiensis* | XP_019337316.1 |
| *Rana temporaria* | *R. temporaria* | XP_040210908.1 |
| *Nanorana parkeri* | *N. parkeri* | XP_018413802.1 |
| *Bufo gargarizans* | *B. gargarizans* | XP_044149601.1 |
| *Xenopus laevis* | *X. laevis* | NP_001011438.1 |
| *Danio rerio* | *D. rerio* | NP_001157466.1 |
| *Oreochromis niloticus* | *O. niloticus* | XP_019218605.1 |
| *Oryzias latipes* | *O. latipes* | NP_001098222.1 |
| *Larimichthys crocea* | *L. crocea* | XP_010742464.1 |
| *Takifugu rubripes* | *T. rubripes* | XP_003968131.1 |
| *Poecilia reticulata* | *P. reticulata* | XP_008394870.1 |
| *Latimeria chalumnae* | *L. chalumnae* | XP_005992709.1 |
| *Protopterus annectens* | *P. annectens* | XP_043922967.1 |
| *Rhinatrema bivittatum* | *R. bivittatum* | XP_029447888.1 |
| *Carcharodon carcharias* | *C. carcharias* | XP_041045390.1 |
| *Rhincodon typus* | *R. typus* | XP_020391815.1 |
| *Homo sapiens* | *H. sapiens* | NP_001032752.1 |

**Supplementary Table 3.** The accession numbers of genes used in syntenic analysis.

| **Species** | **Gene** | **Accession ID** | **Species** | **Gene** | **Accession ID** |
| --- | --- | --- | --- | --- | --- |
| Human | *MYC* | NM_001354870.1 | Mouse | *Myc* | NM_001177352.1 |
|  | *FAM49B* | NM_001256763.2 |  | *Fam49b* | NM_001360035.1 |
|  | *ASAP1* | NM_001247996.2 |  | *Asap1* | NM_001276461.1 |
| Chicken | *Myc* | NM_001030952.2 | Lizard | *Myc* | XM_028736154.1 |
|  | *Fam49b* | XM_040690973.1 |  | *Fam49b* | XM_028736152.1 |
|  | *Asap1* | XM_025148178.2 |  | *Asap1* | XM_028736147.1 |
| American alligator | *Myc* | XM_019481749.1 | Red-eared  Turtle | *Myc* | XM_034763617.1 |
|  | *Fam9b* | XM_006264806.3 |  | *Fam9b* | XM_034763619.1 |
|  | *42Sp50* | XM_019481771.1 |  | *42Sp50* | XM_034763622.1 |
|  | *Asap1* | XM_019481770.1 |  | *Asap1* | XM_034763623.1 |
| Frog | *Myc* | NM_204059.1 | Coelacanth | *Myc* | XM_005992648.2 |
|  | *Fam9b* | XM_031903306.1 |  | *Fam9b* | XM_014486459.1 |
|  | *42Sp50* | NM_001011438.1 |  | *42Sp50* | XM_005992647.2 |
|  | *Asap1* | NM_001097266.1 |  | *Asap1* | XM_014486457.1 |
| Nile tilapia | *Otulina* | XM_003439243.5 | Japanese Medaka | *Otulina* | XM_004081145.4 |
|  | *Ankha* | XM_019363062.2 |  | *Ankha* | XM_004081146.4 |
|  | *Myc* | XM_003439244.5 |  | *Myc* | NM_001308999.1 |
|  | *Fam49b* | XM_019363061.2 |  | *Fam49b* | XM_023950205.1 |
|  | *42Sp50* | XM_019363060.2 |  | *42Sp50* | NM_001104752.1 |
|  | *Asap1* | XM_005448981.4 |  | *Asap1* | XM_023950200.1 |
|  | *Nfx1* | XM_005448987.4 |  | *Nfx1* | XM_023950311.1 |
|  | *Smarcd3b* | XM_005448990.4 |  | *Smarcd3b* | XM_023950319.1 |
| Stickleback | *Otulina* | XM_040166712.1 | Fugu | *Otulina* | XM_011608022.2 |
|  | *Ankha* | XM_040166711.1 |  | *Ankha* | XM_003968080.3 |
|  | *Myc* | XM_040167711.1 |  | *Myc* | NM_001037875.1 |
|  | *Fam49b* | XM_040167492.1 |  | *Fam49b* | XM_011608026.2 |
|  | *42Sp50* | XM_040167490.1 |  | *42Sp50* | XM_003968082.3 |
|  | *Asap1* | XM_040167485.1 |  | *Asap1* | XM_029842512.1 |
|  | *Nfx1* | XM_040167489.1 |  | *Nfx1* | XM_011608030.2 |
|  | *Smarcd3b* | XM_040167772.1 |  | *Smarcd3b* | XM_029842519.1 |
| Large yellow croaker | *Otulina* | XM_027274018.1 | Guppy | *Otulina* | XM_008396657.1 |
|  | *Ankha* | XM_027274209.1 |  | *Ankha* | XM_008396655.2 |
|  | *Myc* | NM_001303373.1 |  | *Myc* | XM_008396652.2 |
|  | *Fam49b* | XM_019271674.2 |  | *Fam49b* | XM_008396649.2 |
|  | *42Sp50* | XM_010744162.3 |  | *42Sp50* | XM_008396648.2 |
|  | *Asap1* | XM_019271643.2 |  | *Asap1* | XM_008396644.2 |
|  | *Nfx1* | XM_019271676.2 |  | *Nfx1* | XM_008396641.2 |
|  | *Smarcd3b* | XM_019271682.2 |  | *Smarcd3b* | XM_017301934.1 |
| Northern pike | *Otulina* | XM_010885545.4 | Spotted gar | *Otulina* | XM_006634362.2 |
|  | *Ankha* | XM_013139538.4 |  | *Ankha* | XM_015354438.1 |
|  | *Myc* | XM_010885537.4 |  | *Myc* | XM_006634360.2 |
|  | *Fam49b* | XM_010885536.4 |  | *Fam49b* | XM_015354492.1 |
|  | *42Sp50* | XM_010885535.5 |  | *42Sp50* | XM_006634359.2 |
|  | *Asap1* | XM_010885533.5 |  | *Asap1* | XM_015354443.1 |
|  | *Nfx1* | XM_029116432.2 |  | *Nfx1* | XM_015354495.1 |
|  | *Smarcd3b* | XM_013139527.4 |  |  |  |
| Zebrafish | *Otulina* | NM_213142.1 |  |  |  |
|  | *Ankha* | NM_001030259.2 |  |  |  |
|  | *Myc* | NM_131412.1 |  |  |  |
|  | *Fam49b* | NM_207078.1 |  |  |  |
|  | *42Sp50* | NM_001163994.1 |  |  |  |
|  | *Asap1* | XM_009297312.3 |  |  |  |
